# RESTRICTIONS ON RESIDENT CONTACT IN ASSISTED LIVING DURING COVID-19: A MIXED-METHODS STUDY

**DOI:** 10.1093/geroni/igac059.1380

**Published:** 2022-12-20

**Authors:** Lindsay Peterson, Sara Hackett, Kallol Kumar Bhattacharyya, Debra Dobbs

**Affiliations:** University of South Florida, Tampa, Florida, United States; University of South Florida, Tampa, Florida, United States; University of South Florida, Tampa, Florida, United States; University of South Florida, Tampa, Florida, United States

## Abstract

Efforts to protect assisted living residents from COVID-19 by limiting contact warrant attention. Assisted living was developed as a social model where care is provided in a home-like environment. Given the social dimensions of assisted living, we sought to better understand the effects of COVID-19-based restrictions in assisted living. We surveyed (online) assisted living community (ALC) administrators (N=130) between October 2020 and March 2021 as part of a larger project on COVID-19 in Florida. We then interviewed a subset of participants (N=26). Administrators of chain-affiliated ALCs (compared to non-chain) were 2.7 times more likely to report resident-contact limitations had disrupted care (p=0.02). Larger ALCs (25 or more beds) were marginally more likely to report care disruptions (p<0.10). Three main themes emerged from our qualitative interviews – varying interpretation of COVID-19 guidelines, effect of precautions on residents, assisted living as a home. Policy implications of these findings will be discussed.

